# Micro-Energy Harvesting and Self-Powered Sensing for Intelligent Road: A Comprehensive Review

**DOI:** 10.34133/research.1171

**Published:** 2026-03-16

**Authors:** Guiping Zheng, Chao Xing, Lei Zhang, Hengyu Li, Tinghai Cheng, Yiqiu Tan

**Affiliations:** ^1^ School of Transportation Science and Engineering, Harbin Institute of Technology, Harbin 150000, China.; ^2^ Beijing Institute of Nanoenergy and Nanosystems, Chinese Academy of Sciences, Beijing 101400, China.

## Abstract

The sustained growth in global road mileage, combined with rising public expectations for improved travel safety and comfort, has created an urgent demand for sensing technologies in road systems. Nevertheless, conventional monitoring methods are increasingly insufficient for modern intelligent roads (IRs), which represent next-generation infrastructure requiring efficient and sustainable sensing solutions. Existing sensor systems that rely on traditional power sources or cabled power supply suffer from inherent limitations, including short operational lifespans and high maintenance costs. At the same time, current road micro-energy harvesting technologies struggle to meet the demands of large-scale distributed sensor networks in IR, underscoring the critical need for self-powered sensing solutions designed for IR. This review identifies the power supply bottleneck inherent in distributed sensor networks of IR, provides a critical comparison of potential solutions with a focus on micro-energy harvesting technologies, and offers a comprehensive review of the practical applications of self-powered sensors (mainly piezoelectric nanogenerators and triboelectric nanogenerators) within road systems. Furthermore, it proposes forward-looking recommendations and developmental prospects for the field, thereby offering theoretical support to advance the large-scale implementation of self-powered sensing in IR systems.

## Introduction

The accelerating pace of technological advancement and escalating public expectations for transportation services have rendered traditional road systems, designed solely for basic traffic functions, increasingly inadequate to satisfy modern transportation networks’ sophisticated demands [1]. This inadequacy has elevated intelligent roads (IRs) as a transformative infrastructure solution. IRs represent the next-generation infrastructure that synergistically combines advanced materials, information systems, and technologies while maintaining basic transportation functions and exhibiting intelligent capabilities such as energy harvesting and conversion, multidimensional self-perception, environmental self-regulation, and multimodal interaction [2]. Notably, IR’s operational efficacy relies on 2 critical technological foundations: sustainable energy harvesting systems and high-precision sensing networks.

As 2 fundamental pillars of national economic development, transportation and energy sectors demonstrate critical synergies through smart integration initiatives—a transformative pathway for achieving sustainable transportation advancement. The global transportation sector, which accounts for 26.2% of total industrial energy consumption [3], has garnered attention for the potential of renewable energy applications amidst the growing scarcity of nonrenewable resources [4]. The global road network currently spans over 21 million km and continues expanding, with China’s infrastructure representing a particularly important case study. As of the end of 2024, China’s highway system has reached 5.4904 million km in total length, comprising 5.2701 million km (97.3%) of grade 4 or above highways while accommodating 12.1224 million operational vehicles [5]. Under conservative estimation based on standardized 2-lane configurations (3.75 m width per lane), this infrastructure represents a minimum surface area of 4.1 × 10^10^ m^2^ capable of substantial solar energy absorption. Moreover, continuous vehicular movement generates significant interactions at the tire–road and vehicle–environment interfaces. These interactions give rise to diverse high-entropy energy forms, including vibrational, thermal, and intermittent low-grade wind energy [6]. While transportation infrastructure offers extraordinary energy utilization potential, the irregular and discontinuous nature of road-system energy poses significant collection challenges for these high-entropy sources, making advanced micro-energy harvesting technologies indispensable for effective exploitation [7,8].

The rapid global expansion of road networks has precipitated explosive growth in demand for road self-perception systems (encompassing traffic safety and structural health monitoring), driving an exponential increase in sensor network node density [9,10]. However, conventional energy harvesting technologies, such as photovoltaic pavement and wind power generation, are inadequate in meeting the sophisticated power supply requirements of distributed sensor networks in the context of IR construction. While photovoltaic and wind power generation technologies have achieved practical implementation in specific road infrastructure applications such as highway service areas and tunnels [11], they exhibit substantial technical and operational constraints. These limitations manifest in 2 primary dimensions: spatially, the technologies face substantial limitations due to road space availability, particularly in densely populated urban areas [12]; technologically, photovoltaic pavement systems confront multiple challenges including long-term power generation stability, mechanical durability under traffic loads, techno-economic viability, and commercial profitability [13]. Meanwhile, wind-based solutions are constrained by inherent limitations such as high environmental sensitivity and restricted power generation capacity. These compounded technical challenges highlight the pressing need to develop innovative, efficient, and reliable self-powered sensing solutions specifically designed for modern road networks.

**Fig. 1.**
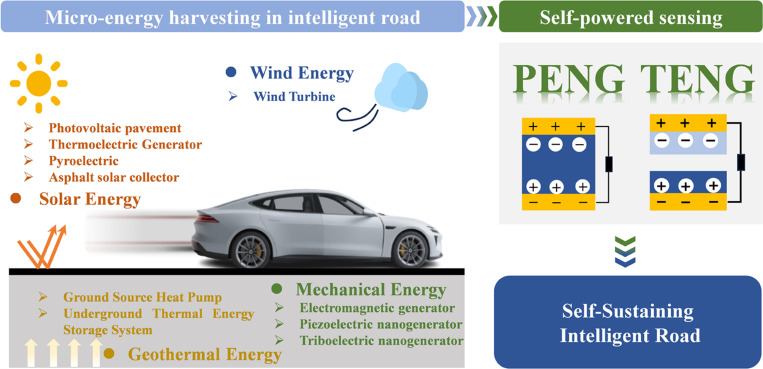
Road energy harvesting and self-powered sensing technologies: A pathway to self-sustaining intelligent roads.

Consequently, this review analyzes the power supply bottlenecks in distributed sensor networks for IR, critically compares the available solutions with a focus on micro-energy harvesting technologies, and specifically examines piezoelectric nanogenerators (PENGs) and triboelectric nanogenerators (TENGs), which represent the 2 mechanical energy harvesting technologies demonstrating the potential for integrated sensing and energy functions. Subsequently, it provides a comprehensive review of the specific applications of both self-powered sensing technologies within road systems, proposes actionable recommendations for future research, and concludes with a forward-looking perspective on the field’s developmental trajectory. Figure 1 illustrates the evolutionary pathway from micro-energy harvesting to self-sustaining intelligent roads, corresponding to the logical framework of this review.

## Dilemma and Solution of Distributed Energy Supply for IR

### Requirements and challenges: Power supply bottlenecks for distributed sensing

It is essential to understand the actual performance of infrastructure during construction and service phases to ensure the safety and service quality of road and transportation systems. This requirement has led to the widespread application of sensing technologies in road system monitoring. Presently, monitoring demands are primarily focused on 2 key areas: the structural health of road infrastructure and the characteristics of traffic flow, relying on various sensors to accomplish data acquisition, processing, and feedback. As illustrated in Table 1, the perception system of IR constitutes a complex, multilevel, and multitask integrated framework.

**Table 1.** Perception system of IR

Perception layer	Core tasks	Typical devices/methods	Deployment characteristics
Infrastructure perception layer (embedded)	Structural health, material condition, and internal response	Strain gauges, fiber Bragg gratings, piezoelectric sensors, temperature and humidity sensors, intelligent aggregates, etc.	Embedded within the structure, with the widest distribution and largest quantity
Dynamic perception layer (on-road and mobile)	Vehicle trajectory, speed, flow rate, model, weight, etc. Detect anomalies on roads	1. Road embedded type: induction magnetic ring, piezoelectric ring array; 2. Roadside equipment: microwave radar, ultrasonic and acoustic sensor systems, infrared systems, laser radar, video image detectors, etc.; 3. Wide area data: photogrammetric acquisition and processing, video and audio analysis; 4. Vehicle data: Vehicle GPS/OBU; sensors like accelerometers, gyroscopes, LiDAR, cameras that have been made available on vehicles	1. Shallow embedded/surface attached; 2 and 3. Installed on the roadside or above, with a wide field of view; 4. Mobility, data come from vehicles.
Roadside environment and service perception layer (roadside)	Panoramic monitoring, meteorological environment, traffic signs, communication relay, etc.	High-definition pan tilt zoom camera, weather station, variable information sign, 5G micro base station, etc.	Centralized deployment along the line side, with key locations but relatively few quantities, and high equipment volume and power consumption.

The sensing nodes of the IR system can be categorized into 3 tiers according to deployment location, core task, and maintainability: (a) the infrastructure-embedded perception layer, where sensors are integrated within infrastructure to monitor physical parameters, primarily including kinematic metrics (e.g., displacement and acceleration), mechanical properties (e.g., force, deformation, stress, and strain), and environmental characteristics (e.g., humidity and temperature) [14,15]. In recent years, micro-electromechanical systems (MEMS) technology has been increasingly applied in road infrastructure monitoring. Owing to their advantages of low power consumption and compact size, MEMS-based sensors have progressively replaced certain conventional types, leading to the emergence of low-power devices such as smart aggregates and MEMS accelerometers [16,17]. (b) Dynamic perception layer, through which dynamic information is acquired by means of embedded, roadside, wide-area, and floating vehicle-based methods. Fixed-point monitoring employs piezoelectric arrays and induction loops embedded in the pavement, or radar and video equipment installed roadside, to obtain traffic parameters. Wide-area monitoring perceives the status of regional road networks via video analytics and related technologies and could be powered by roadside electrical grids. Mobile monitoring utilizes platforms such as floating vehicles and unmanned aerial vehicles to collect dynamic data [18]. (c) Roadside environment and service perception layer, where devices are characterized by high power consumption, centralized deployment, limited quantity, fixed locations, and the capability to be connected to the power grid.

To explicitly demonstrate the energy consumption characteristics of sensing units, several typical sensors and their respective power consumption are summarized in Table 2.

**Table 2.** Typical small sensors and their power consumption in road systems

Monitored object	Perceptual parameter	Typical sensors	Power consumption
Environmental characteristics	Temperature	Thermocouples	0.5 μA to 30 mA
		Thermistors	1 to 80 mA
	Humidity	Capacitive sensors	2 μA to 4 mA
		Resistive sensors	0.5 μA to 5 mA
Kinematic metrics	Acceleration	Accelerometer	≤20 mA
	Displacement	LVDT displacement sensor	≤20 mA
	Attitude	Gyroscope	≤80 mA

Although the power consumption of a single sensor node can be as low as the microwatt level, the dense deployment of nodes in road networks, often reaching tens to hundreds per kilometer, leads to considerable cumulative energy demand and poses significant challenges regarding overall power supply costs. Currently, lithium-ion batteries, characterized by high energy density and mature manufacturing technology, have been adopted as the primary power source for micro-sensors, typically providing a cycle life ranging from 300 to 5,000 cycles and a self-discharge rate between 10% and 15% [19]. However, when applied to large-scale distributed road sensing networks that are often buried and require long-term operation, several inherent limitations of lithium-ion batteries become apparent. These include a finite cycle life, performance degradation under harsh environmental conditions such as temperature and humidity fluctuations as well as mechanical vibration, and high maintenance and replacement costs over the system’s full life cycle. Consequently, such batteries often fail to meet the requirements for long-term operational sustainability, economic feasibility, and reliable power supply in these scenarios. This indicates a structural contradiction between the conventional battery-based power supply model and the fundamental needs of IR distributed sensing networks in 3 key aspects: total life-cycle cost, operational and maintenance feasibility, and scalability for large-scale deployment.

### Solution: Multidimensional comparison of micro-energy harvesting technologies

**Fig. 2.**
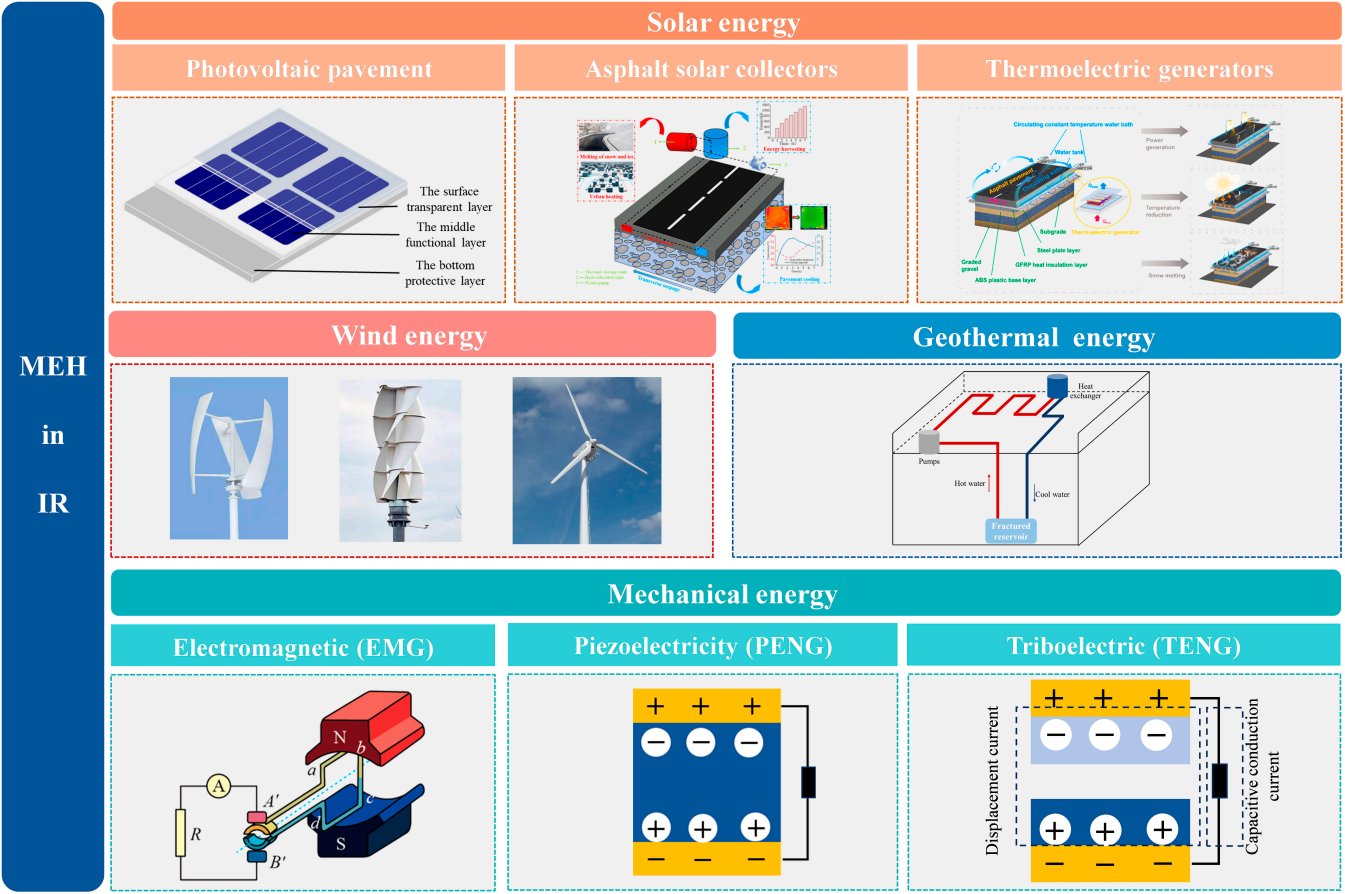
The study provides a comparative assessment of various energy harvesting technologies applicable to road systems, evaluating them in terms of power density or energy conversion efficiency, levelized cost of energy (LCOE), and technology readiness level (TRL), as summarized in Table 3. LCOE is often used as a metric to rank the competitiveness of power generation technologies. LCOE is estimated based on the ratio of the total project life-cycle cost and the total lifetime energy production. The TRL ranges from 1 to 9, where 1 is the lowest level of readiness and 9 is the highest. The following levels are included: Level 1 represents the clarification of fundamental principles, level 2 denotes the proposal of technical solutions, level 3 represents the verification of key technologies, level 4 signifies the formation of functional units, and level 9 means the achievement of batch application [97, 98].

The development of renewable energy harvesting technologies offers a novel pathway to address the energy needs of road systems. The road system constitutes a complex multielement coupled system that integrates 3 fundamental components: road infrastructure, traffic participants, and environmental elements, while simultaneously harboring diverse forms of renewable energy. These energy sources can be systematically classified into 4 primary categories based on their generation mechanisms: solar energy, wind energy, geothermal energy, and mechanical energy. Correspondingly, a diversified suite of micro-energy harvesting technologies has been developed, including photovoltaic pavement (PV pavement), thermoelectric generators (TEGs), asphalt solar collectors (ASCs), micro wind turbines, ground-source heat pumps (GSHPs), electromagnetic generators (EMGs), piezoelectric energy harvesters (PENGs), and TENGs, as illustrated in Fig. 2.

The study provides a comparative assessment of various energy harvesting technologies applicable to road systems, evaluating them in terms of power density or energy conversion efficiency, levelized cost of energy (LCOE), and technology readiness level (TRL), as summarized in Table 3. LCOE is often used as a metric to rank the competitiveness of power generation technologies. LCOE is estimated based on the ratio of the total project life-cycle cost and the total lifetime energy production. The TRL ranges from 1 to 9, where 1 is the lowest level of readiness and 9 is the highest. The following levels are included: Level 1 represents the clarification of fundamental principles, level 2 denotes the proposal of technical solutions, level 3 represents the verification of key technologies, level 4 signifies the formation of functional units, and level 9 means the achievement of batch application.

**Table 3.** Comparison of energy harvesting technologies in IR

Categories	Source	Principle	Power density	Energy conversion efficiency	LCOE ($/kW h)	TRL
Wind turbine	Wind energy	Wind → mechanical energy → electrical energy	32.50 W/m^2^ [102] (Data were collected at 10 stations in Malaysia over a 10-year period.)	23.2% [103] (The wind harvester, which was installed at the entrances and exits of a real tunnel, had S-rotors and H-rotors built into it.)	0.0817 [104] (Onshore wind farm in the United States)	9
GSHP	Geothermal energy	Underground temperature difference → heat exchange → thermal/electrical energy	–	20%–50% [105] (Data were collected from 24 GSHP projects in China.)	0.38–0.58 [106] (A shallow geothermal site in Hungary)	9 [107]
PV pavement	Solar energy	Photovoltaic effect (solar energy → electrical energy)	8.9 W/m^2^ [108] (SR bike lane has 54 polycrystalline silicon modules and 80 wafer-based cells, located at 52.494°N, 4.7666°E.)	50%–70% [23] (The results obtained after analyzing the component photovoltaic efficiency loss of solar pavement.)	1.65 to 7.95 [23] (The results obtained after analyzing the projects of solar pavement.)	9
TEG		Seebeck effect (temperature difference → electrical energy)	2 W/m^2^ [109] (The output voltage of RTEGS was about 0.4 V by asphalt mixture slab 300 mm × 300 mm by size.)	~42% [110] (The system was built with cement concrete [1.2 m × 0.6 m] and hydronic pipe [outer diameter is 20 mm, and the inner diameter is 16 mm] imbedded in it.)	0.9 [26] to 95.74 [25] (Two-TEG prototypes [64 mm × 64 mm])	3
ASC		Absorption of pavement structure heat → fluid circulation → thermal energy	–	20%–70% [111] (Utilizing the proper pipe layout boosts the thermal efficiency by up to 70%.)	0.15 [25] to 4.21 [112] (SERSO system in Swiss with piping under pavement.)	3
EMG	Mechanical energy	Electromagnetic induction (mechanical vibration → electrical energy)	1,277.16 W/m^3^ [113] (At a frequency of 9 Hz and a displacement of 3.0 mm, 1.34 × 10−4 m^3^.)	–	278.95 [112] (Vibration-based system installed on bridge cables)	4
PENG		Piezoelectric effect (mechanical stress → electrical energy)	32.1 W/m^2^ [114] (Four piezoelectric transducers, 3–12 Hz, 0.2–0.7 mm, 18 × 18 cm^2^.)	–	2.32 [115] to 106.97 [27] (Cymbals can be embedded in the Florida roadway network, and the LCOE is varied from $35.66 to $106.97/kWh for 5–15 years.)	4
TENG		Contact electrification + electrostatic induction (friction → electrical energy)	20.96 W/m^3^ [116] (Device had a height of 3.7 in. and a diameter of 1.5 in.)	–	–	3

As indicated in Table 3, current micro-energy harvesting technologies for road system applications demonstrate considerable variation across key performance indicators. Although wind power generation exhibits a relatively low LCOE ($0.0817/kWh), its economic viability largely depends on large-scale commercial deployment (TRL 9). In integrated road settings, the installation of micro-wind turbines is often constrained by the spatially limited environments of urban roads and tunnels, where they are typically mounted on roadside medians or central dividers. Furthermore, the wind resources available in such systems are characterized by significant turbulence and irregularity, which present ongoing challenges to achieving consistent energy conversion efficiency. GSHP systems have been widely adopted internationally for many years and are well-recognized as effective solutions for heating and cooling applications [20]. Balbay and Esen [21] conducted the first systematic investigation into the practical feasibility of utilizing GSHPs for snow and ice melting on sidewalks and bridge decks, concluding that the application yielded promising operational outcomes. However, during the design process of GSHP systems, several critical factors, including drilling space and depth, fluid velocity within buried pipes, and backfill materials are frequently overlooked, each of which can significantly affect the operational stability of the systems [22]. Consequently, the full life-cycle economic feasibility of deploying GSHP technology in road environments remains a subject requiring further verification.

In comparison with conventional photovoltaic power generation systems, PV pavements are subject to more complex and severe operational conditions, including traffic loads, dust accumulation, and associated factors, which collectively result in a substantially shorter actual service life compared to the projected lifespan for most projects. Additionally, the LCOE of PV pavements ranges from $1.65/kWh to $7.95/kWh [23], which typically prevents such systems from achieving investment recovery over their life cycle. Given that electricity from fossil fuels still costs about $0.20/kWh [24], researchers argue that PV pavements will only become economically viable when their LCOE drops below this $0.20/kWh threshold [13,23]. As a typical shallow pipe-pavement energy harvesting system, ASC captures and stores thermal energy from road surfaces, thereby contributing to the mitigation of the urban heat island effect while enabling applications such as winter road de-icing and building heating. Because of the absence of deep excavation or drilling required by conventional GSHP systems, its LCOE can be as low as $0.15/kWh [25]. Nevertheless, several significant limitations restrict its widespread adoption: the system depends on an external power supply to circulate fluid within the pipes; its performance is closely tied to pavement temperature and declines under low-temperature or low-sunlight conditions; and conventional road maintenance operations, including milling, pose a risk of damage to the buried piping network. The conversion of thermal energy from road surfaces into electricity is facilitated by TEGs, which function by directly transforming temperature gradients between heat sources and radiators into electrical energy based on the Seebeck effect. The efficiency of these generators is a pivotal factor in determining their power generation capacity. At present, the power output of TEGs remains relatively low; for example, under a temperature difference of 350 °C, the CMO-25-42S module produces only 1.37 W [26]. In an evaluation of TEG application potential across the entire Florida road network, Guo and Lu [27] estimated that if covered with TEG systems, the network could harvest up to 55 GWh of electricity daily, corresponding to an LCOE of approximately $2.31/kWh.

Compared with the aforementioned technologies, which primarily adhere to the “environmental energy to power supply” pathway, EMG, PENG, and TENG technologies based on mechanical energy harvesting exhibit a distinct development logic. Despite their current low TRL and high LCOE, these technologies directly derive energy from active components of the road system, such as pedestrians and vehicles, and typically deliver outputs on the order of several hundred milliwatts, sufficient to power most low-consumption road sensors. More importantly, they demonstrate significant advantages in system integration: their compact form allows them to be embedded or surface-mounted within road systems, eliminating the need for large-scale infrastructure modifications. Consequently, they offer superior practicality compared to conventional energy harvesting technologies, particularly in deployment convenience, minimal infrastructure interference, and scalability. It is noteworthy that, although traditional energy harvesting technologies such as wind, photovoltaic, and geothermal systems have achieved economic viability in the macro-scale renewable energy sector, their energy sources compel reliance on large-scale energy storage units. Moreover, these technologies adhere to the conventional electricity model of “centralized generation and distributed distribution”. Therefore, powering thousands of sensors dispersed across a road network would necessitate a comprehensive microgrid along with the accompanying deployment of complex infrastructure for power conversion, management, and distribution [28–30], a requirement that presents significant engineering and economic challenges. Therefore, these technologies are applicable to powering centralized roadside equipment. In contrast, distributed sensing networks in IR systems exhibit a fragmented demand profile, with each low-power node requiring merely microwatt- to milliwatt-level power. By integrating the 3 stages of energy harvesting, conversion, and utilization within the same physical location and device, namely, through the implementation of self-powered sensors, a viable pathway emerges to address the aforementioned challenges.

## Applications of Self-Powered Sensing Technologies in IR

In recent years, PENGs and TENGs have demonstrated considerable application potential in IR systems owing to their superior self-powder sensing capabilities. Piezoelectric self-powered sensors predominantly employ inorganic piezoelectric ceramics, such as lead zirconate titanate (PZT), which exhibit excellent linear response and stability under normal stress and vibration. As a result, PENG-based self-powered sensors show distinct advantages in embedded, mechanically sensitive applications, including structural health monitoring and weigh-in-motion systems. TENGs operate on the principles of contact electrification and electrostatic induction. Their high flexibility in material selection and structural design makes them particularly suitable for scenarios involving vehicle–road interaction and wearable monitoring, both of which are critical to the coordinated evolution of IR systems toward “human–vehicle–road” integration. Accordingly, this section details the application progress of both technologies in IR systems. The “PENG-based self-powered sensors in IR” section focuses on the application of PENGs in structural embedding and quantitative sensing, while the “TENG-based self-powered sensors in IR” section systematically elaborates on the cutting-edge advances of TENGs in diverse scenarios, including vehicle interaction, wearable devices, and roadside environmental perception, leveraging their unique functional attributes.

### PENG-based self-powered sensors in IR

Considerable research has been conducted on PENG-based self-powered sensors within IR infrastructure, with the current academic focus predominantly directed toward 3 critical domains: real-time traffic characteristic monitoring, structural health assessment, and intelligent compaction for asphalt pavement.

#### Traffic characteristic monitoring

PENG-based self-powered sensors have been integrated by researchers either within pavement structures or into intelligent tire systems to enable multifunctional traffic monitoring. These applications include traffic flow assessment, dynamic vehicle weighing, speed detection, vehicle type classification, and tire–road contact analysis. Yang et al. [31] employed PZT-5H piezoelectric ceramic sensors for traffic dynamic weighing applications. Khalili et al. [32] constructed an energy-efficient dynamic weighing system utilizing PZT elements capable of simultaneously monitoring multiple vehicle parameters, including velocity, axle count and spacing, axle load distribution, and vehicle classification. Jiang et al. [33] proposed a comprehensive perception calculation methodology for vehicle speed, axle count, wheelbase, and total vehicle weight determination based on piezoelectric sensor arrays, culminating in the development of an integrated perception calculation system. To further optimize sensor performance, substantial research has been directed toward the development of advanced piezoelectric composite materials with enhanced electromechanical properties. Toroń et al. [34] developed a piezoelectric sensor using SbSI/epoxy composite material, investigating the correlation between vehicle speed and sensor output voltage/generated energy. Liang et al. [35] fabricated a novel piezoelectric sensor from polyvinylidene fluoride/lead zirconate titanate/carbon nanotubes (PVDF/PZT/CNTs) composites, achieving simultaneous energy harvesting from vehicular motion and real-time monitoring of axle load, speed, and wheelbase parameters. Aishwarya et al. [36] engineered a self-powered speed sensor based on PVDF/Li-AA piezoelectric films, where temporal differential analysis enabled load-independent vehicle speed detection. The most innovative advancement emerged from He et al. [37], who successfully transformed conventional asphalt into piezoelectric asphalt with integrated sensing capabilities. This functionalized pavement material captures tire–pavement interaction data without compromising structural integrity and calculates key traffic parameters like speed and wheelbase, promoting a shift from passive infrastructure to intelligent, responsive systems.

As the critical vehicle–pavement interface, tires present challenges for wired sensor systems. The development of intelligent tire systems based on PENG wireless self-powered sensors has emerged as a pivotal innovation for enhancing vehicle safety. These systems enable comprehensive monitoring of key tire parameters including pressure, deformation, wheel loading, frictional characteristics, and tread wear patterns, thereby improving the reliability of both tires and their associated control systems [38]. Nguyen et al. [39] investigated the energy harvesting capability of PVDF-based self-powered sensors from tire deformation, with field tests on asphalt and concrete pavement surfaces confirming their potential for detecting tire–road contact parameters. Wu et al. [40] developed a smart tire prototype incorporating PVDF piezoelectric sensors, which achieved precise vertical load measurements by accounting for critical variables such as contact patch length, vehicular speed, and tire pressure. Sun et al. [41] further advanced this field by proposing an intelligent tire system equipped with piezoelectric sensors, where comprehensive analysis of sensor signal responses under varied slip angles, loads, tire pressures, vehicle speeds, slip ratios, and tread wear conditions during tire rolling enabled the development of a novel tire slip angle estimation methodology.

The development of PENG-based self-powered sensors in traffic monitoring has been comprehensively demonstrated across 2 primary dimensions: vehicle-integrated and road-embedded systems. In the latter approach, sensors are directly embedded within the road infrastructure, with the objective of enabling intelligent management at the macroscopic traffic flow level. Conversely, the smart tire solution is dedicated to the real-time acquisition and feedback of vehicle state parameters, thereby enhancing active safety and handling performance.

#### Structural health monitoring

Piezoelectric self-powered sensors have been extensively deployed for pavement structural health monitoring, primarily focusing on 4 critical areas: pavement surface roughness assessment, hidden crack identification, structural fatigue damage monitoring, and mechanical response/deformation detection. Concerning pavement surface irregularity analysis, Quan et al. [38] engineered a PVDF-based piezoelectric smart tire, subsequently establishing a friction coefficient prediction model that achieved 94.86% prediction accuracy. Yang et al. [42] designed an intelligent tire system using piezoelectric cables for pavement condition sensing. By integrating proprietary algorithms for roughness estimation and anomaly detection, this system improved the real-time performance and accuracy of pavement monitoring. In the domain of pavement defect detection, Alavi et al. [43] developed a spherical piezoelectric smart aggregate to detect fatigue damage in asphalt pavement structures. Through comprehensive analysis of piezoelectric response signal characteristics, various damage states, particularly crack propagation, can be effectively identified. Ji et al. [44,45] conducted a systematic investigation into the acoustic attenuation properties of piezoelectric smart aggregates (SPA), establishing a quantitative relationship model between crack width and acoustic parameters. Furthermore, they elucidated the influence patterns of environmental factors (temperature and humidity) and material parameters (aggregate particle size and porosity) on monitoring outcomes, thereby laying a theoretical foundation for the precise identification of cracks. In the field of structural mechanics response monitoring, Otto et al. [46] employed piezoelectric sensors to collect pavement deformation data under vehicular loads, successfully predicting the deflection of typical flexible asphalt pavements and thereby providing a novel methodology for assessing the load-bearing capacity of such pavements. Differing from previous studies, Gu et al. [47] embedded piezoelectric sensors in unbound granular base/subbase layers. They systematically validated the sensors’ ability to monitor dynamic soil stress (vertical and horizontal components) under complex traffic loads, considerably expanding the application of PENG technology to deeper pavement structure monitoring. Cai et al. [48] embedded piezoelectric sensors in subgrade layers to measure output voltage, subsequently performing back-calculation of the subgrade modulus. Their work identified distinct relationships between peak voltage at different depths and the subgrade modulus under varying traffic loads and speeds, demonstrating the considerable potential of this method for health monitoring of transportation infrastructure.

In summary, the technical feasibility of PENG-based self-powered sensors for structural health monitoring of road has been systematically validated by existing research across multiple critical dimensions, including pavement roughness assessment, hidden crack detection, structural fatigue damage monitoring, and the detection of mechanical responses from the pavement surface through to the subgrade. These collective efforts delineate a coherent technical trajectory that advances from surface condition sensing to deep mechanical inversion and from qualitative damage identification to quantitative parameter analysis, thereby demonstrating the versatile application potential of this technology in comprehensive road health monitoring.

#### Intelligent compaction detection of asphalt pavement

Compaction represents one of the most pivotal processes in asphalt pavement construction, with the compaction degree serving as a key quality control indicator. Traditional testing methodologies, exemplified by core sampling techniques, exhibit inherent limitations including temporal lag and structural invasiveness. The advent of PENG-based self-powered sensors has effectively addressed these challenges by providing an innovative, real-time and nondestructive monitoring alternative that enables continuous quality assessment throughout the compaction process. Vennapusa et al. [49] applied static piezoelectric cone technology to control granular pavement compaction. By embedding piezoelectric earth pressure cells in the base layer, they enabled simultaneous measurement of vertical and horizontal stresses during vibratory roller operation. Their research elucidated the stress field distribution patterns under varying compaction conditions, thereby establishing quantitative metrics for compaction quality assessment. Dan et al. [50] focused on particle motion dynamics during compaction, employing piezoelectric smart aggregates to investigate internal dynamic responses. Through systematic analysis, an intrinsic relationship between particle responses and asphalt mixture compaction characteristics was established, offering theoretical support for compaction process optimization. Wang et al. [51] comprehensively reviewed PENG self-powered sensors in intelligent compaction (IC) systems, highlighting 3 innovative sensor types: SmartRock for monitoring changes in aggregate orientation, fiber Bragg grating arrays for distributed strain field measurement, and integrated circuit piezoelectric accelerometers for capturing vibration response characteristics.

The application of PENG-based self-powered sensors to asphalt pavement compaction monitoring signifies a paradigm shift, transitioning from post-construction assessment to real-time, intelligent control during the construction process. This advancement enables multiscale monitoring, spanning from macro-level stress–strain analysis down to the aggregate motion within asphalt mixtures.

**Fig. 3.**
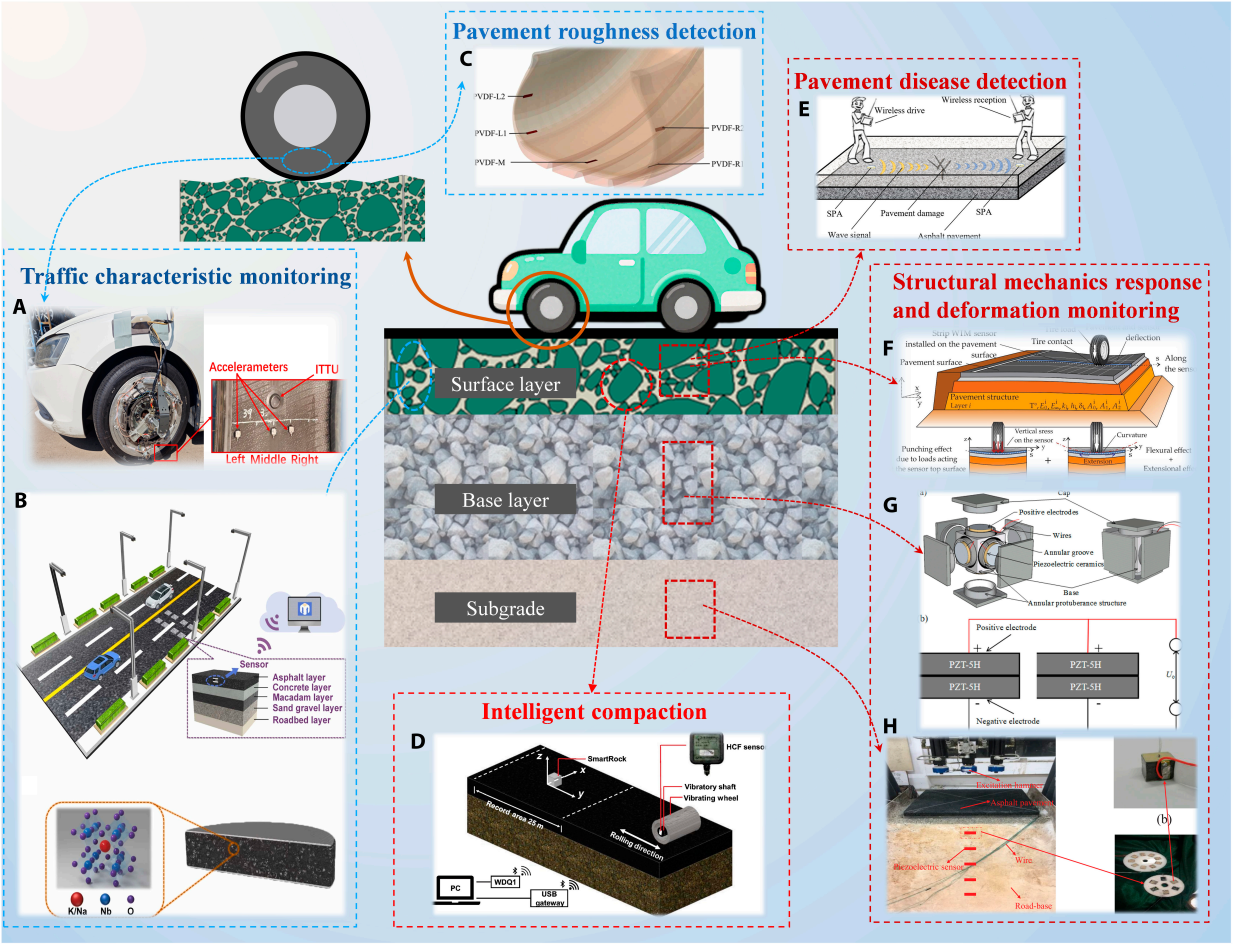
Application of PENG-based self-powered sensors in IR. (A) Vertical load estimation techniques for intelligent tire systems. (B) The concept of transforming asphalt from a pavement structural component to a sensing component was presented. (C) Estimating the tire–road friction coefficient using an intelligent tire system and brush tire model. (D) Monitoring the dynamic response of asphalt pavement under vibration compaction load using SmartRock sensors. (E) Self-powered aggregate for detecting cracks in asphalt pavement. (F) Comprehensive response of piezoelectric sensors to load and road deflection effects. (G) Piezoelectric transducer to monitor dynamic soil stress in unbound granular materials of road engineering. (H) A real-time monitoring method for Road-Base Quality (MRBQ) based on a soil dynamic model and piezoelectric sensors buried in road base. Representative structures based on Refs. [37,38,40,44,46–48,50].

A visual analysis of the literature (Table 4 and Fig. 3) indicates that despite the application research on PENG-based self-powered sensors in road systems in IR showing a diversified trend, and the ability of some devices to achieve outputs in the hundreds of volts under specific excitation, with a power of 27 mW and a power density of 1,000 mW/m^2^, existing studies exhibit deficiencies in characterizing key performance metrics. Specifically, there is insufficient quantitative analysis and reporting of device energy conversion efficiency or power output. Moreover, experimental validation and data on the long-term durability of materials and devices under simulated real-world conditions, including traffic loads and coupled multifield environmental effects, remain notably inadequate. These characterization blanks to some extent limit the assessment of its engineering applicability and full life-cycle performance.

**Table 4.** Application of PENG-based self-powered sensors in IR

Deployment method	Materials	Monitored parameters	Voltage (V)	Power (mW)	Power density (mW/m^2^)	Durability (cycles)
Embedded in the surface layer of the road	SbSI/epoxy [34]	Traffic flow parameters	1.15	27	–	–
	PVDF/PZT/CNTs [35]		9.85	72.3 × 10^−6^	–	500,000 (0.7 MPa, 10 Hz)
	PVDF/Li-AA [36]		100	–	1,000	–
Material of the road itself	Asphalt [37]		2.5–14.7	–	–	10,000 (0.7 MPa, 2.5 Hz)
Tire	PVDF [38]	Pavement friction coefficient	1–10	–	–	–
Embedded in the road	PZT-4 [43]	Pavement damage	~1	–	–	–
	PZT/PVDF [44]	Hidden cracks in road	~75	–	–	–

### TENG-based self-powered sensors in IR

#### Road-mounted sensors

With the development of Intelligent Transportation System (ITS), it has been demonstrated that TENG-based self-powered sensors exhibit broad application prospects in the field of IR, where their application scenarios and functionalities are highly diverse. From a technical implementation standpoint, 3 predominant deployment modalities have emerged for road-mounted TENG-based self-powered sensors.

The integration of TENG with conventional road infrastructure has demonstrated remarkable functional extensibility. Through the synergistic incorporation of TENG-based self-powered sensors into existing road infrastructure, these hybrid systems not only preserve the inherent functionality of traditional infrastructure but also acquire advanced intelligent monitoring capabilities. A representative implementation was developed by Heo et al. [52], who engineered a TENG-incorporated speed bump system that effectively transforms conventional road infrastructure into a multifunctional, self-powered unit capable of simultaneous vehicle speed detection and warning generation. This design maintained the original purpose of speed bumps while achieving the dual goals of speed monitoring and energy harvesting, embodying the multifunctional use concept in intelligent transportation.

Functional integration solutions for road surface have been demonstrated to exhibit more flexible and diverse technical characteristics. Among these solutions, the spray-painting-type TENG for pavement surface offers the advantages of simple construction and large-scale deployment, which makes it possible to achieve intelligent monitoring across the entire road area. Yun et al. [53] proposed the paint-based TENG (PBT) and subsequently implemented an intrusion detection system capable of accurately identifying traffic violations. Attached-type TENGs have achieved notable results in traffic safety monitoring and adaptability to road service environment through material selection and modification, structural design, and the utilization of different working modes. For instance, Mishra et al. [54] designed a unique road safety sensor system by employing high-efficiency printed TENGs, which is used to control the movement of ascending and descending vehicles on sharp turns and one-way roads in hilly areas. Wang et al. [55] developed a gradient-structured integrated pressure–velocity sensor (PVS), in which the tilted dielectric electrode intermediate layer and gate design markedly reduce data fluctuation errors while enhancing the sensitivity of synchronous monitoring of traffic flow and vehicle speed. Luo et al. [56] reported a DMT-sensor based on the SE mode and proposed a speed measurement method using this DMT-sensor. The result showed that even under adverse road conditions, the DMT-sensor could achieve an accuracy of over 80%.

Road-embedded TENG-based self-powered systems have been successfully implemented by integrating sensors within the layers of pavement structures, thereby enabling high-precision monitoring of traffic parameters. Li et al. [57] developed an intelligent traffic monitoring system capable of continuous all-weather operation by combining self-healing piezoresistive sensors with TENG. This system demonstrated exceptional capability in accurately detecting instantaneous vehicle velocities, determining accident liability, and monitoring vehicular loads, while simultaneously enhancing sensor durability and operational reliability to ensure sustained pavement monitoring performance. Of particular note is the groundbreaking work by Ji et al. [58], who pioneered a subgrade-embedded energy harvesting system incorporating suspended-layer TENG arrays. Their self-powered intelligent connected transportation system (SP-ICTS) features a strategic design: terminal units act as sensing nodes, while 8 central units function as energy harvesting modules, collectively forming an on-site microgrid to power roadway sensors. This integrated system achieved 5 essential functionalities: autonomous power generation, precise positioning, real-time sensing, wireless communication, and intelligent guidance.

In order to systematically and comprehensively evaluate the current application status of road-mounted TENG-based self-powered sensors in the field of traffic safety monitoring, a comprehensive review and analysis of the existing road-mounted TENG sensors are conducted in this review. As is presented in Table 5, the key technical parameters of various sensors are summarized in detail, encompassing the material of the tribo-layer, working mode, output performance, and specific monitoring parameters. From the perspective of the technical implementation mechanism, the current road-mounted TENG sensors are primarily based on 2 typical working modes, namely, CS and SE. Through sophisticated multiphysics coupling designs integrated with advanced data processing algorithms, these systems have successfully achieved 3 principal functionalities: (a) high-precision real-time monitoring of vehicular dynamic parameters (e.g., velocity, load distribution, and trajectory patterns); (b) intelligent identification of potential traffic hazards (such as regulatory violations); and (c) optimized traffic flow management. In terms of voltage output, existing research demonstrates a wide range, from several volts to hundreds of volts, which reflects the considerable influence of different materials, structures, and operating modes. However, most studies fail to report corresponding values for power or power density, thereby limiting systematic quantification and comparison of their practical energy conversion capabilities. Furthermore, the characterization of durability remains largely confined to laboratory conditions, often citing parameters such as applied force, frequency, and cycle count, for instance, up to 100,000 cycles at 10 Hz, alongside associated output stability data. These technological breakthroughs have provided an essential hardware foundation and technical support for the construction of ITS, thereby demonstrating the unique application value and remarkable development potential of TENGs in the field of traffic safety monitoring. Future progress in materials science and IoT integration is anticipated to further enhance the operational performance and expand the application domains of TENG-based self-powered sensors.

**Table 5.** Application of road-mounted TENG-based self-powered sensors in monitoring traffic characteristics

Deployment		Name	Material	Working mode	Monitored parameters	Voltage (V)	Power (mW)	Power Density (mW/m^2^)	Durability (cycles)
Integrated with road infrastructure		SB-TENG [52]	PVC	SE/FS	Self-powered warning system, vehicle speed	49.8	–	–	–
Functionalization of pavement surface	Spray-painting	PBT [53]	Paint spray	SE	Violation of traffic regulations by vehicles	25.4	–	17.6	–
	Attached	CN-STS [117]	PET, PVDF-CNT	CS	Overspeed capture, overlap, and license plate recognition	3.3	–	–	–
		E-TENG, S-TENG [118]	PA, PI	FS	Vehicle speed monitoring and overspeed warning	150	42	–	–
		TB-TENG [119]	PP, PS	CS	Vehicle speed	184	–	151	6,000 (14.3 N)
		GMS-PVA-TENG [120]	GMS-PVA	SE	Track or warn of traffic violations	150	–	106	7,000
		TND [54]	OHP, ZnS NSs	CS	Vehicles driving at sharp turns	420	–	3.9	10,500
		PVS [55]	PTFE	CS	Vehicle speed	425.98	–	–	10,800 (13 N, 3 Hz)
		DMT-sensor [56]	PTFE	SE	Vehicle speed, acceleration, and direction of motion	150	–	–	–
		SP-TENG [121]	PVDF, silk	CS	Vehicle overload and traffic flow	648	–	24 × 10^6^	10,000 (5 N, 2.5 Hz)
		TENG [99]	NTO/PDMS CF	CS	Vehicle speed and direction finding	195	–	2,150	10,000 (9 N, 5 Hz)
		TENG [122]	PCL-MoS_2_, Nylon-66	CS	Vehicle speed	127	0.022	–	–
		TENG [123]	EVA-PZT-PANI	SE	Vehicle overload and traffic flow	17.8		3	1,200 (5 N, 1 Hz)
		FRP-TES [100]	FRP	SE	Vehicle speed, acceleration	41.6	–	–	10,000
Embedded in the pavement structure		TENG [57]	PPC	SE	Instantaneous speed and load capacity	3	–	–	–
		ml-TENG [124]	Kapton	FS and LS	Vehicle speed	7.2	–	–	–
		TENG [101]	FEP, PA66	CS	The flow of pedestrians and motorcycles on sidewalks, as well as the speed and direction of motorcycles on nonmotorized lanes	590	–	–	–
		RTE harvester [58]	FEP, POM	CS	Vehicle speed and load wheelbase	250	16.41	–	100,000 (10 Hz)

#### Vehicle-mounted sensors

##### Structural health monitoring

Although studies on vehicle-mounted TENG-based self-powered sensors for pavement structural health monitoring remain at a nascent stage, considerable application potential has already been demonstrated. Yang et al. [59] deployed stretchable spring-shaped electrode TENGs on vehicle suspension springs, which enabled quantitative assessment of pothole depth and distribution by analyzing vibration amplitude and frequency characteristics during vehicular motion. Subsequently, the research team [60] developed an advanced vehicle-mounted pavement health monitoring system based on spring-guided auxiliary triboelectric sensors (S-TENG), which facilitated real-time detection of surface irregularities including potholes and bumps through chassis-mounted S-TENG arrays. For pavement skid resistance monitoring, Pang et al. [61] designed a textile-inspired TENG smart tire system that combined real-time pavement friction monitoring and hazard through integrated warning mechanisms, markedly improving driving safety. Subsequent innovations by the team [62] yielded a self-powered vehicle–road integrated electronic device featuring a multilevel fractal structure, in which tire-embedded sensors acquired comprehensive pavement condition data. These groundbreaking investigations have established a solid and important foundation for the advancement of vehicle-mounted TENG-based self-powered sensors in pavement structural health monitoring. The core concept of current research on road health monitoring based on vehicle-mounted TENG sensors is to transform vehicles into distributed mobile sensing units, facilitating real-time monitoring of road defects and performance. This represents a advancement in the field of road health monitoring, surpassing the limitations of traditional fixed-point monitoring methods.

##### Traffic safety monitoring

Vehicle-mounted TENG-based self-powered sensors have exhibited remarkable versatility in intelligent driving applications, primarily enabling comprehensive driving behavior monitoring and vehicular status assessment through nonintrusive deployment strategies [63]. From a functional standpoint, TENGs located at different positions within the vehicle each exhibit unique characteristics. Based on their deployment locations, the applications of these sensors can be broadly classified into the following categories. For driving behavior monitoring, pedal-mounted TENGs effectively capture foot operations, such as acceleration and braking pattern, and identify hazardous maneuvers like abrupt acceleration or emergency braking, supporting multidimensional safety monitoring [64–67]. Zhang et al. [68] further developed an innovative Driver Training Assistance System integrating 3 triboelectric sensing modules: a gearshift sensor for detecting engagement sequences, a steering angle sensor for measuring wheel rotation, and a pedal sensor for monitoring actuation states. These integrated sensors worked in concert to provide comprehensive data for driver training and behavior analysis, which was of great significance for improving driving safety and efficiency. Notably, Lu et al. [69] designed an intelligent cockpit carpet that utilizes a zoned, sponge-based triboelectric sensor to support human–vehicle interaction in the left-foot area while accurately identifying driving intention in the right-foot area, thereby reducing the driver’s operational load. Complementary to this, seatbelt-integrated TENGs have demonstrated capability in real-time detection of driver positional changes, enabling accurate identification of hazardous behaviors including excessive turning angles and forward leaning [70]. Furthermore, steering wheel-mounted TENGs have been successfully implemented with multifunctional capabilities encompassing continuous monitoring of steering parameters and operational frequency [71,72], reliable detection of hands-off driving conditions [73], and autonomous emergency obstacle avoidance responses [74].

Considerable progress has been made in vehicle operational condition monitoring through the implementation of TENGs. Wheel- and tire-mounted TENG sensors have demonstrated exceptional capability in monitoring real-time tire load distribution, detecting road slope variations, and identifying abnormal tire pressure conditions [75–78]. Furthermore, vehicle environment perception systems integrated with TENGs have been successfully deployed for blind spot monitoring, obstacle detection, and pedestrian recognition, thereby providing critical data support for collision avoidance systems and emergency braking [79,80]. Yang et al. [81] developed an innovative vibration monitoring system based on TENG, which employed advanced pattern recognition algorithms to intelligently distinguish between various operational states, such as normal driving, emergency braking, and collision events.

As is clearly demonstrated in Table 6, the application of TENG-based self-powered sensors in traffic safety monitoring has fully manifested the unique advantages that TENGs possess in enhancing vehicle intelligence and driving safety. Because of their noninvasive characteristics, these sensors are more convenient for practical application deployment. With the ongoing advancement of ITS and IoT technologies, vehicle-mounted TENGs are anticipated to assume an increasingly pivotal role in intelligent driving systems, thereby providing robust technical foundations for the development of next-generation IR.

**Table 6.** Application of vehicle-mounted TENG-based self-powered sensors in monitoring traffic safety

Deployment	Name	Material	Working mode	Monitored parameters	Voltage (V)	Power (mW)	Power density (mW/m^2^)	Durability (cycles)
Pedel	ST-TENG [64]	FEP	FS	The habit of stepping on the brake and accelerator pedals	400	2.4	–	–
	GM-TENG [65]	PTFE	SE	Braking state	20	–	–	72,000 (180 N, 2 Hz)
	TENG [66]	PDMS-SBTO	FS	Normal and abnormal braking	13.5	0.00098	–	600 s (5 N, 1 Hz)
	HPIS [67]	PTFE	CS	Emergency braking, rapid acceleration, normal braking and acceleration	10	–	–	–
Carpet	S-TENG [69]	PDMS, PET	SE	Normal acceleration, rapid acceleration, normal braking, emergency braking, acceleration and braking intent	5	–	–	72,000 (5 N, 2 Hz)
Seat belt	APU-TENG [70]	PI, PTFE, Kraft paper	CS	Driver’s breathing condition, turning direction, and angle	6.98	–	–	–
Steering wheel	SSAS [71]	PTFE	FS	Driver fatigue level	9	–	–	–
	TENG [72]	Kapton	CS	Driver’s steering action	200	–	–	–
	TENG [74]	Nylon, PTFE	CS	Driver’s steering intention	3	–	–	–
	KOS-TS [73]	FEP	CS	Driver’s grip strength	20	–	–	500,000 (1 Hz)
Wheel and tire	RSW-TENG [75]	PTFE, water	FS	Road slope and wheel speed detection	1.68–1.82	–	–	–
	St-TENG [76]	PTFE, PU	CS	Tire load	17.3	–	–	300,000 (20 N, 5 Hz)
	r-TENG [77]	Rubber–asphalt	CS	Crossing the line or deviating from the route	13.3	–	6.48	7,000 (5 N, 1.5 Hz)
Vehicle body	SNC-TENG [79]	MXene	Noncontact SE	Vehicle sentinel mode and blind spot detection	29.9	–	–	5,000 (1.6 Hz)
	LB-TENG [80]	PLBEM	SE	Vehicle surrounding environment	170	–	5	2,000 (2 Hz)
Chassis	EP-Cu/n-GaN TVNG [81]	n-GaN, EP-Cu	–	Vehicle driving status	27	–	4,190	–

##### Monitoring environmental conditions

Vehicle-mounted TENG-based self-powered sensors for road environment monitoring can be systematically classified into 2 principal categories: particulate/powder-based TENG and raindrop-based TENG (see Table 7). Current research indicates that particulate/powder-based TENGs have been predominantly employed in vehicular exhaust particulate filtration and detection applications [82,83], demonstrating considerable potential for environmental remediation. Within IR, raindrop-based TENGs have attracted notable research interest due to their distinctive performance advantages, with primary implementations in intelligent vehicle window systems and automated windshield wiper control [84–86]. Real-time precipitation monitoring is a critical technological requirement for intelligent vehicle development. However, conventional battery-powered rainfall sensors typically exhibit inherent limitations, including insufficient sensitivity, poor environmental adaptability, and weak interference resistance. More importantly, existing systems often lack integrated data analysis capabilities. An ideal self-powered precipitation monitoring system should be capable of real-time rainfall intensity analysis to enable precise wiper speed modulation. Therefore, developing novel raindrop sensors with high sensitivity, wide operational temperature ranges, and self-powering characteristics is of substantial practical significance for next-generation vehicle systems.

**Table 7.** Application of vehicle-mounted TENG-based self-powered sensors in monitoring road environmental conditions

Deployment	Name	Material	Working mode	Monitored parameters	Voltage (V)	Power (mW)	Power density (mW/m^2^)	Durability
Vehicle exhaust pipe	V-TENG [83]	PTFE pellets	CS	PM2.5 collection (self-powered triboelectric filter)	3,000	–	–	–
Wiper	SL-TENG [84]	PTFE	SE	Distinguish rainfall	16.62	0.003	–	–
	TENG [85]	PVDF	SE	Raindrop frequency	67.33	–	–	–
	SCP-TENG [86]	SCP	SE	Sensing rainfall	12.89	–	8.2	–

#### Flexible wearable sensors

Despite the widespread prevalence of dangerous driving behaviors in daily life, such as fatigued driving and mobile phone use, statistical data indicate that they contribute to over 30% of serious traffic accidents, representing a substantial threat to road safety. Existing monitoring technologies, however, are confronted with 3 principal limitations: (a) detection inaccuracies stemming from interindividual physiological variations; (b) compromised recognition reliability under heterogeneous illumination conditions; and (c) the constrained operational endurance characteristic of conventional battery-dependent systems. In this context, wearable flexible TENG-based self-powered sensors have emerged as a highly promising solution in the field of driver state monitoring. As summarized in Table 8, these sensors are typically deployed on the face, neck, hands, or wrists to enable precise tracking of physiological and behavioral signals. Lu et al. [87] achieved multidimensional fatigue and distraction monitoring using facial and cervical TENG patches. Fatigue was assessed via 4 indicators: blink duration, blink interval, percentage of eyelid closure, and yawning frequency, while distraction was identified through mouth movements and head position. In subsequent work [88], the team developed a triboelectric sensing glove that formed an intelligent takeover assistance system for autonomous vehicles. This system dynamically adjusts takeover timing based on real-time driver status, enhancing the safety and stability of the takeover process. Luo et al. [89,90] pioneered a monitoring system combining NaCl/PVA hydrogel neck rings with PEO-PDMS sensors, which facilitated monitoring of neck movement and breathing status, providing quantitative evaluation of fatigue and attention levels. Chen et al. [91] achieved breakthrough performance by integrating electromagnetic–triboelectric wristbands with deep learning algorithms, developing a behavior recognition system that attained 98.21% accuracy in classifying 6 distinct driving behaviors. Wu et al. [92] proposed a magnetic levitation hybrid wristband (MS-HW) system incorporating multiscale convolutional channel attention residual networks, which demonstrated exceptional 97.53% recognition rates, unequivocally validating the transformative potential of TENG in hazardous driving behavior monitoring applications.

**Table 8.** Application of flexible wearable TENG-based self-powered sensors in monitoring driver status

Deployment	Name	Material	Working mode	Monitored parameters	Voltage (V)	Power (mW)	Power density (mW/m^2^)	Durability (cycles)
Face	PL-TENG [87]	PDMS, skin	SE	Fatigue and distraction	200	–	350	10,000
Neck	NH-TES [89]	NaCl/PVA, skin	SE	Driver’s neck movement	49.21	–	–	–
	PP-TENG [90]	PDMS, PEO	CS	Driver’s fatigue and concentration level	18.52	–	14	5,000 (31.11 kPa, 1 Hz)
Hand	F-TENG [88]	Ecoflex	–	Nondriving behavior	8.57	4.59	–	–
Wrist	ET-WSS [91]	PTFE	–	Driving behavior	0.507	0.678	26.78 W/m^3^	10,000 (3 Hz, 3 cm)
	MS-HW [92]	PTFE	FS	Driving behavior	0.1859	0.39	–	–

#### Roadside sensors

Wind-based TENG sensors have been demonstrated to exhibit unique advantages in wind speed monitoring [93–95]. Particularly noteworthy is the adaptive rotary triboelectric–electromagnetic hybrid wind energy harvester (SAREH) developed by Li et al. [96], which ingeniously utilized TENG output to actuate dust removal mechanisms while employing EMG to power particulate detection instrumentation, thereby establishing a fully self-sustaining system for air purification and quality monitoring. It was shown in this research that not only did it offer new insights into the design of efficient energy harvesting systems, but it also opened up new avenues for the development of self-sustaining environmental monitoring systems.

Table 9 presents a summary of the current applications of roadside TENG-based self-powered sensors in road environment monitoring. Although research in this field is still in its infancy and the existing literature is limited, the published research indicates that TENGs possess pronounced technical advantages and exhibit great application potential in road environmental monitoring.

**Table 9.** Application of roadside TENG-based self-powered sensors in monitoring road environmental conditions

Deployment	Name	Material	Working mode	Monitored parameters	Voltage (V)	Power (mW)	Power density (mW/m^2^)	Durability (cycles)
Roadside	W-TENG [93]	Hydrogel	SE	Wind speed and wind pressure	6	0.0008	–	–
	GS-TENG [94]	PTFE	SE	Dusty	130	–	–	–
	Flag-type TENG [95]	PVDF, PA6	SE	Wind speed	150	–	11.14	2,800

**Fig. 4.**
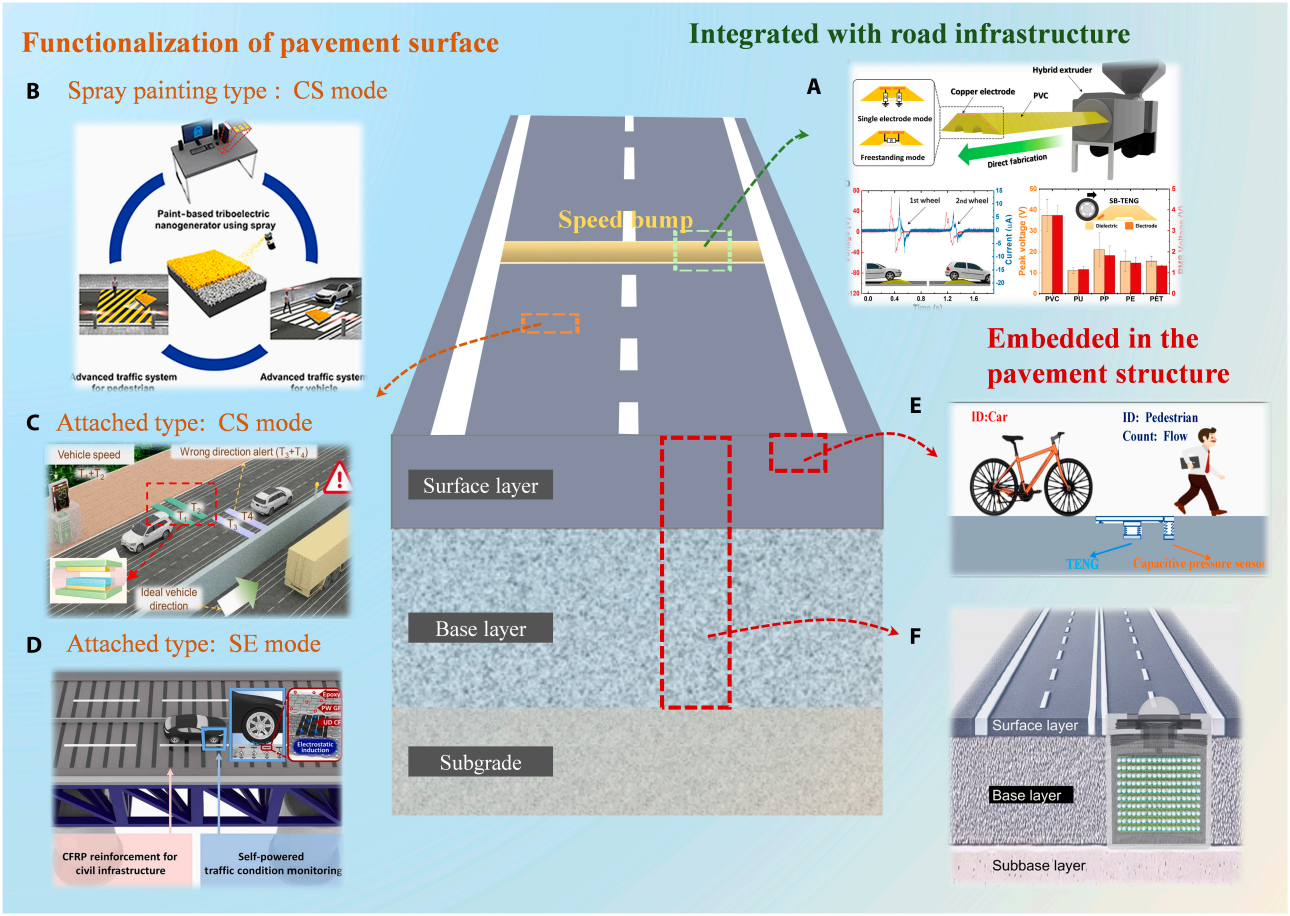
Road-mounted TENG-based self-powered sensors for monitoring traffic characteristics. (A) Triboelectric speed bump as a self-powered automobile warning and velocity sensor. (B) Paint-based triboelectric nanogenerator toward smart traffic system and security application. (C) TENG was utilized as a sensor to find vehicle speed and wrong direction alerts. (D) Self-powered triboelectric sensor for efficient traffic monitoring. (E) A self-powered real-time wireless traffic monitoring system based on TENG. (F) Roadbed tribological energy harvester. Representative structures based on Refs. [52,53,58,99–101].

**Fig. 5.**
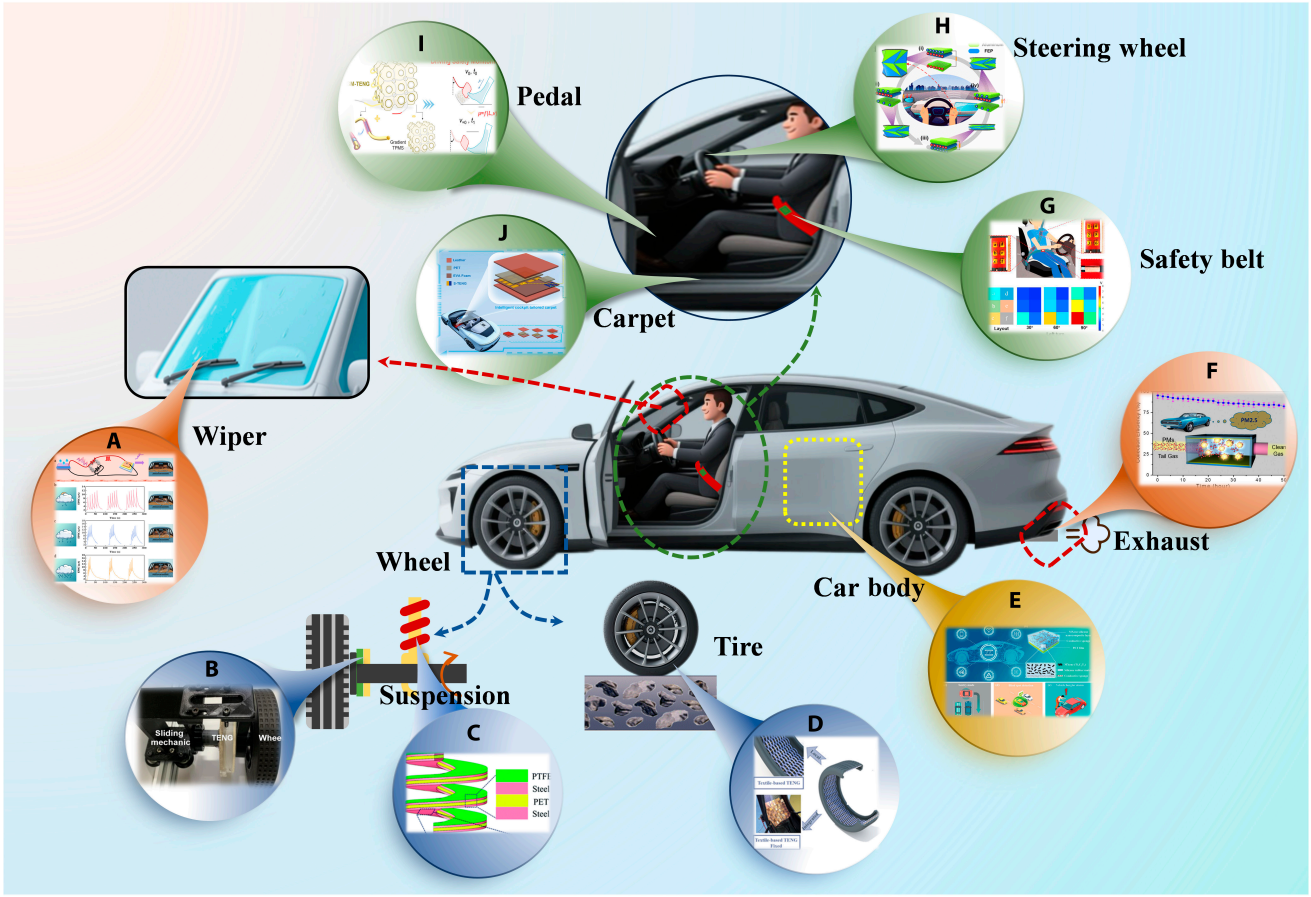
Vehicle-mounted TENG-based self-powered sensors in IR. (A) Self-powered intelligent water droplet monitoring sensor. (B) A rotational switched-mode water-based TENG for vehicle monitoring. (C) Spring-like-electrode TENG for sensing road potholes. (D) Textile-inspired TENG as self-powered skid resistance sensor. (E) Self-powered noncontact triboelectric sensors and applications in intelligent vehicle perception. (F) Removal of particulate matter emissions from a vehicle using a self-powered triboelectric filter. (G) A self-powered smart safety belt enabled by TENG for driving status monitoring. (H) Monitoring driving behavior using TENG on the steering wheel. (I) A natural gradient biological-enabled multimodal TENG for driving safety monitoring. (J) An intelligent cockpit tailored carpet for human–vehicle interaction enhancement and driving intention recognition. Representative structures based on Refs. [59,61,65,69,70,73,75,79,83,85].

**Fig. 6.**
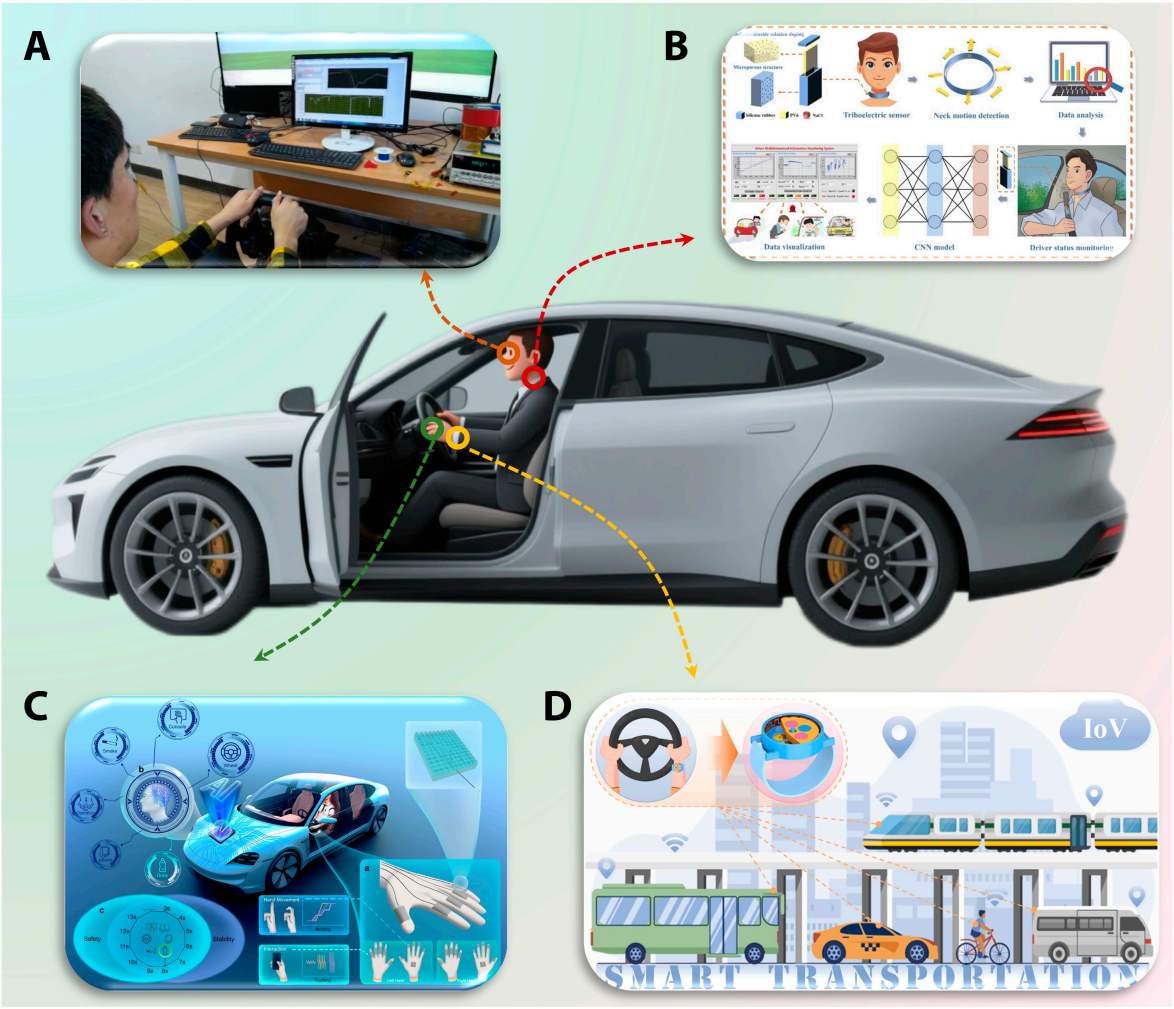
Application of TENG-based flexible wearable sensors in monitoring driver status. (A) TENG as a self-powered sensor for driver fatigue and distraction monitoring. (B) Triboelectric sensors for driver multidimensional information monitoring. (C) Triboelectric sensor gloves for real-time behavior identification and takeover time adjustment. (D) Driver behavior detection system for smart transportation. Representative structures based on Refs. [87–89, 91].

Similarly, a systematic review and visualization of the relevant research on triboelectric self-powered sensors (Tables 5 to 9 and Figs. 4 to 6) indicate that, in comparison with PENG-based self-powered sensors, TENG-based self-powered sensors exhibit considerably greater diversity in material selection, device configuration, working modes, and application scenarios, with their output voltage spanning from several volts to several kilovolts and their power reaching the milliwatt level. However, existing studies also exhibit notable limitations in characterizing key performance metrics. Although some literature reports durability data indicating cycle lifetimes between approximately 2,000 and 500,000 cycles, most related testing has been conducted under simplified laboratory conditions, such as the application of single-digit Newton-level forces and low-frequency periodic excitation. However, in terms of actual road conditions, it is evident that the weight of a small car is approximately equivalent to that of 1.5 tons, whereas a large car weighs in the range of 4.5 tons, thereby exceeding the pressure range that was subject to testing. It is important to acknowledge that, given the marked differences between the test conditions and the random and high-intensity mechanical load that typify real-world road environments, the actual service life of sensors under realistic operating conditions is likely to be considerably shorter than the values reported in laboratory studies. To elucidate this discrepancy more precisely, actual traffic parameters are introduced for estimation: taking a typical national highway with an average daily traffic volume of 50,000 vehicles, among which heavy-duty trucks constitute 15%, each sensor is subjected to thousands of random, high-intensity mechanical impacts daily. A passenger car weighs approximately 1.5 tons, whereas a heavy-duty truck weighs about 4.5 tons, both far exceeding the pressure ranges examined in existing literature. The instantaneous stress and excitation frequency produced by a single heavy-duty truck passage can be equated to dozens or even hundreds of cycles under standard laboratory testing conditions. Furthermore, such descriptions do not sufficiently incorporate the coupled effects of multiple environmental factors, such as temperature and humidity variations, prevalent in real-world road conditions, thereby failing to accurately reflect the performance degradation patterns and ultimate service life of devices under actual operating conditions. Consequently, under the complex load of actual road conditions, the equivalent service life of the sensor may be reduced to merely a few days, which is markedly lower than the theoretical values derived from laboratory data.

## Conclusion

This article systematically examines the energy supply challenges confronting distributed sensing networks in IR systems, conducts a multidimensional comparison and critical evaluation of road micro-energy harvesting technologies, and provides a comprehensive review of the specific applications of self-powered sensing technologies within IR. While the majority of the past reviews focused on energy harvesting pavements in general, this study centers on analyzing their practical suitability and adaptability for distributed sensing energy supply. The major findings concluded from this comprehensive literature review are as follows:

1. Traditional road energy harvesting technologies hold considerable value within the energy transition, and their large-scale deployment has demonstrated both effectiveness and economic viability in centralized energy supply scenarios. However, their applicability encounters major challenges when extended to distributed road sensing networks.

2. PENG-based self-powered sensors have been successfully deployed in 3 key domains, namely, traffic parameter identification, structural health monitoring, and road construction quality control, which collectively demonstrate their integrated technical capability spanning from energy harvesting to information perception. TENGs, leveraging their material and structural flexibility, exhibit broad application potential in areas such as vehicle–road interaction sensing, wearable safety monitoring, and ambient micro-energy harvesting.

3. Current research still faces several critical limitations: Primarily, most reported results remain confined to laboratory or small-scale experimental stages. The long-term durability of piezoelectric and triboelectric materials under realistic road conditions, including cyclic traffic loads and coupled temperature-humidity effects, has not been comprehensively validated. Consequently, signal stability and operational lifespan continue to represent core bottlenecks for engineering implementation. Secondly, existing research predominantly focuses on sensing performance under single-parameter or idealized conditions, lacking systematic validation in scenarios involving multifield coupling, complex traffic flow, and full life-cycle operation. It should be noted that both TENG and PENG outputs are in the form of alternating-current pulses. Whether serving as energy harvesting units or self-powered sensors, their operational cycle follows a “generate–store–utilize” sequence, which subsequently activates sensors or data-transmission modules. Therefore, the transition from discrete devices to reliable systems continues to face challenges related to packaging integration, signal decoupling, and power-consumption management. Furthermore, although piezoelectric smart pavement materials, such as piezoelectric asphalt, have enabled the functionalization of the pavement material itself, their preparation processes, cost-effectiveness, and compatibility with existing road construction systems remain subjects requiring comprehensive assessment.

## Summary and Prospects

### Recommendations for future work

Despite rapid advances in self-powered sensing technology, its path toward large-scale deployment in road system remains impeded by a series of challenges, including insufficient long-term durability and reliability under the coupled influence of severe traffic loads and environmental conditions; the absence of standardized packaging and integration solutions that accommodate diverse infrastructures and sensing requirements; and an incomplete framework for power management, signal processing, and system-level reliability assessment in the transition from laboratory prototypes to engineered field nodes.

Therefore, to advance self-powered sensing technology from laboratory research to large-scale engineering implementation, focused and collaborative breakthroughs are required in the following 3 key directions:

• High-performance materials for road systems: It should focus on novel functional composites that exhibit high electro-mechanical conversion efficiency, excellent mechanical durability, and long-term environmental stability, while also exploring feasible pathways for directly endowing sensing and energy harvesting functions into bulk road materials such as asphalt mixture or cement concrete, with the aim of achieving the design objective of sensors possessing a service life equivalent to that of the road infrastructure itself.
• Integration and deployment methods of intelligent microsystems: A highly integrated, self-sustaining intelligent sensing microsystem should be developed, incorporating efficient mechanical energy harvesting, ultra-low power signal processing, and reliable short-/long-range wireless transmission modules. Simultaneously, standardized and modular technologies for large-scale deployment and networking must be established to enable the transition from discrete functional devices to stable and reliable perceptual systems.
• Full life-cycle verification: Comprehensive, full life-cycle performance verification and reliability assessment, which spans from materials and devices to integrated systems, must be conducted to provide robust support for the engineering application and industrial promotion of the technology.

Through interdisciplinary collaboration encompassing materials, device, engineering, and information technology, self-powered sensors are positioned to serve as the nerve endings of next-generation IR, thereby advancing the transformation of road infrastructure into self-sensing, adaptive, and sustainable systems.

### Perspectives

**Fig. 7.**
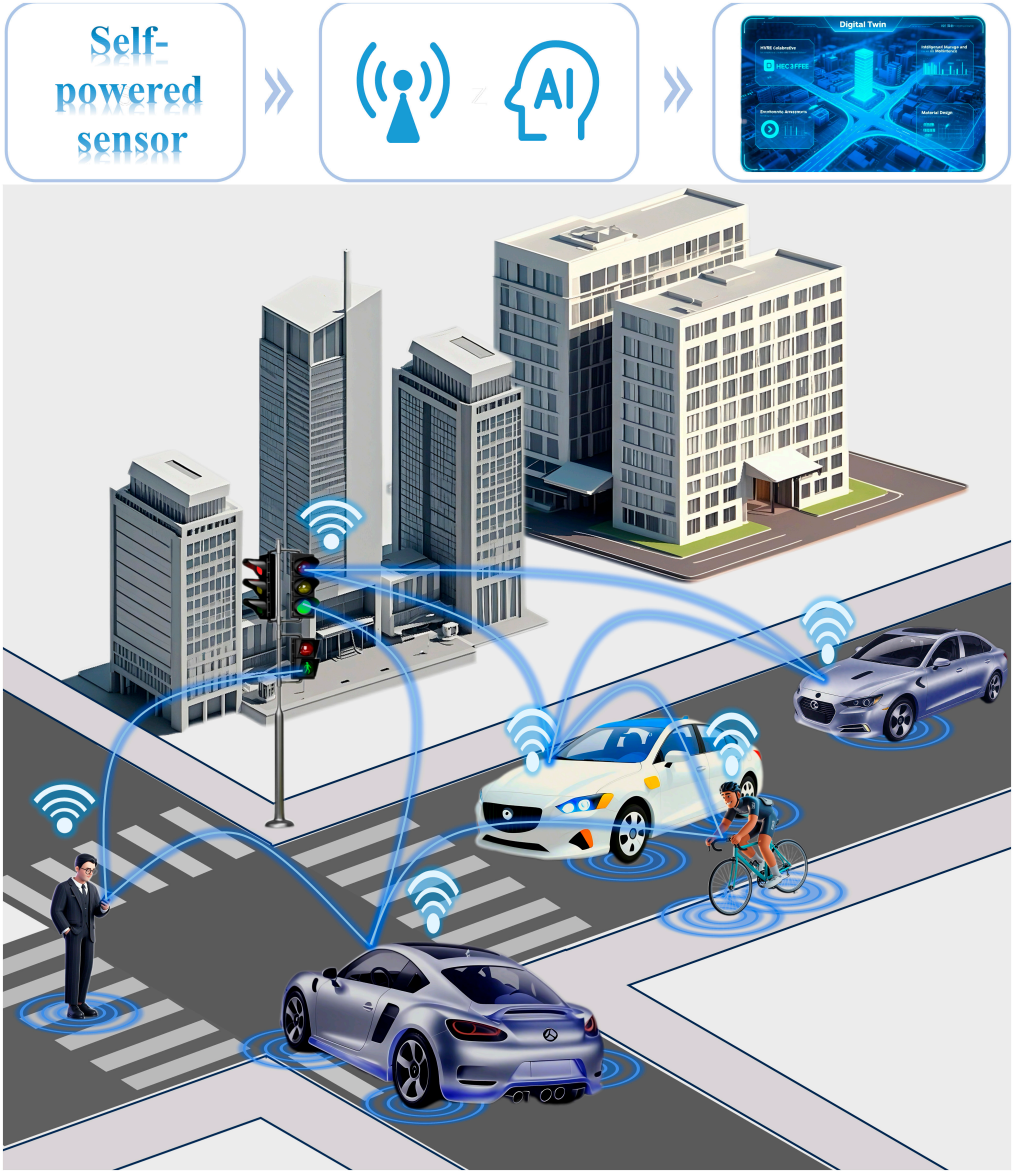
Self-powered intelligent connected transportation system.

The progressive integration of artificial intelligence, big data analytics, and IoT technologies has facilitated widespread adoption of PENG- and TENG-based self-powered sensing systems in IR. Although these technologies are still confronted with challenges such as long-term durability, standardization protocols, and large-scale deployment feasibility, their prospects are becoming increasingly promising. Supported by synergistic 5G communication networks and edge computing technologies, these self-powered sensing technologies are driving the evolution of multidimensional collaborative ecosystems encompassing human, vehicles, roads, and environment, which establish critical foundations for advancing next-generation SP-ICTS, as shown in Fig. 7.

The core objective of SP-ICTS lies in establishing distributed microgrid networks through advanced road micro-energy harvesting technologies, thereby fundamentally transforming conventional transportation infrastructure from passive carriers into intelligent systems capable of simultaneous energy harvesting and bidirectional data exchange, which can effectively address the current challenges related to energy sustainability and notably enhance the intelligence level of transportation systems. Specifically, the operational framework encompasses 3 principal dimensions: (a) self-powered sensors harvest energy derived from vehicle–road–environment interactions, simultaneously fulfilling sustainable energy objectives and enabling real-time data acquisition; (b) multidimensional datasets incorporating environmental parameters, pavement structural conditions, and traffic dynamics provide robust support for traffic safety operations and management; and (c) synergistic integration of efficient energy harvesting and advanced data analysis capabilities facilitates the evolution of SP-ICTS into comprehensive systems featuring integrated self-powering, sensing, positioning, communication, and navigation capabilities. Such technological integration endows road with powerful real-time data processing and autonomous decision-making functionalities, ultimately optimizing traffic operational efficiency, ensuring driving safety, and propelling the transportation sector toward intelligent and sustainable development paradigms.

## Data Availability

The data used in this review document are based on the respective literature works. All data are available.
